# The Impact of Coping Skills in Post-traumatic Growth of Healthcare Providers: When Mental Health Is Deteriorating Due to COVID-19 Pandemic

**DOI:** 10.3389/fpsyg.2021.791568

**Published:** 2021-12-03

**Authors:** Lulejete Prekazi, Vjosa Hajrullahu, Shegë Bahtiri, Blerta Kryeziu, Blertë Hyseni, Besarta Taganoviq, Florim Gallopeni

**Affiliations:** ^1^Heimerer College, Prishtine, Kosovo; ^2^Faculty of Medicine, Department of Nursing and Midwifery, University of Prishtina, Pristina, Kosovo

**Keywords:** post-traumatic growth, coping, mental health, healthcare providers, COVID-19

## Abstract

**Background:** Frontline healthcare providers are consistently exposed to potentially traumatic events while assisting patients with COVID-19. Post-traumatic growth (PTG) happens when a person can transform trauma and use adversity in one’s advantage. In response to limited studies on positive outcomes that may occur from the pandemic; this study aimed to elucidate the positive impact of coping with COVID-19 outbreak on mental health, such as PTG.

**Methodology:** The study comprised a sample of 691 healthcare providers 59% female, including physicians (*n* = 138) and nurses (*n* = 550), working in public health facilities in Kosovo, with an average age of 41.6 years (*SD* = 10.79). They were asked to complete a questionnaire with four parts: Socio-demographic, GHQ-28, COPE and PTGI. A deterioration of mental health with somatic symptoms leading to the escalation due to COVID-19 outbreak was found.

**Results:** Female healthcare providers reported more clinical symptoms as well as higher coping skills scores than men. The domains in which positive changes were most frequently observed were Relating to Others, New Possibilities and Personal Strength. There was no significant direct effect of mental health on PTG in the mediation model, though a significant indirect effect was observed for coping skills.

**Conclusion:** The results suggest that levels of mental health exacerbation do not play a conclusive role in determining levels of PTG, as long coping mechanisms are in place. The development and implementation of interventions to minimize COVID-19-related mental health consequences, by fostering PTG among healthcare providers could be highly beneficial in pandemic response work.

## Introduction

The global spread of coronavirus disease 2019 (COVID-19), resulting in the World Health Organization (WHO) declaring COVID-19 as a pandemic, has become a medical serious concern across more than 200 countries worldwide (Wang et al., 2020; World Health Organization [WHO], 2020). The rapid spread of the COVID-19 resulted in many deaths, mostly due to respiratory problems. Kosovo has been affected by the pandemic since March 13, in 2020 when the first COVID-19 cases were confirmed. By December 22, 2020, in the time when the study was initiated, in Kosovo there were 49,169 confirmed cases of COVID-19, 173,145 suspected and tested and 1,262 deaths (National Institute of Public Health [NIPH], 2020). To restrain the spread of the virus, restrictions in local and international travel, in-house isolation or quarantine were the most common responses and measures enforced by the governments worldwide (Chopra et al., 2020).

Recent studies provide evidence of unprecedented psychological impact of COVID-19 on healthcare providers engaged in the diagnosis, treatment, and care for patients with COVID-19 due to restrictions and the work under stressful conditions (lack of hospital facilities, fear of contagion and spreading the virus, working over-time, wearing personal protection equipment— PPE-, restraining from food, drinks, and the toilet during working hours due to PPE, and more) while managing critical issues daily (Barello et al., 2020; Gupta et al., 2020; Kalaitzaki et al., 2020; Lai et al., 2020). Frontline healthcare providers are continuously reporting increasing levels of anxiety (Neto et al., 2020), associated with functional impairments, alcohol or drug coping, symptoms of depression like extreme hopelessness, and passive suicidal ideation (Lee, 2020). Approximately 50% of physicians have reported poor sleep (Zhang et al., 2020) due to contagious nature of the disease and emergency nature of their work during COVID-19 pandemic (Qiu et al., 2020).

Having been infected with COVID-19 infection and being exposed to risk in work settings, frontline healthcare providers are consistently exposed to potentially traumatic events while assisting patients with COVID-19 (Wu et al., 2019; Kalaitzaki et al., 2020) involving a threat to life and mental health in daily basis, a major upheaval of their life habits linked to confinement and the difficulty of projecting themselves into the future (Constant, 2020). Work-related trauma can act as a catalyst for positive post-trauma changes (Wu et al., 2019) involving “life-changing” psychological shifts in thinking and relating to the world that is deeply meaningful (Tedeschi and Moore, 2021). Although the negative psychological impact of COVID-19 on mental health have been examined in recent studies, little is known about the potential positive outcomes that may occur from the pandemic (Kalaitzaki et al., 2020). Despite the adversities of trauma, some individuals report positive life changes, known as post-traumatic growth (PTG), including an increased appreciation for life, meaningful interpersonal relationships, sense of personal strength, changed priorities, and a richer existential/spiritual life (Tedeschi and Calhoun, 1995, 1996, 2004; Rogan et al., 2013), as a result of their cognitive efforts to deal with challenging life events (Tedeschi and Calhoun, 2004, 1995). A systematic review found that 53% of individuals who endure trauma have experienced PTG from having to cope with life stressors (Wu et al., 2019), enjoying greater psychological and even physical wellbeing (Manning-Jones et al., 2015).

In addition, adaptive coping strategies may be a way to foster PTG in healthcare providers (Rodríguez-Rey et al., 2017). Coping strategies refer to behavioral and cognitive efforts that help to reduce the influence of a stressful condition and are used when its demands exceed individual resources (Girma et al., 2021). Significant cognitive structures nullified by the traumatic events for purposefully thinking about the trauma were found to play an important role to construct the worldview (Yang and Ha, 2019). According to Tedeschi and Calhoun (2004), the coping strategies used to manage stressors are significant in the development of PTG. It is also suggested that PTG mutually interacts with life wisdom and the development of the life narrative, and that it is an ongoing process, not a static outcome. A meta-analysis conducted by Prati and Pietrantoni (2009) showed that, of the many stress coping strategies, positive reappraisal and religious coping have the largest effect on PTG. The same was found in other studies, emphasizing meaning-focused coping strategies, in particular positive reappraisal among cancer patients (Sears et al., 2003) and HIV/AIDS population (Siegel et al., 2005). Conversely, avoidance coping strategies hinder the probability of growth after trauma (Helgeson et al., 2006).

To the best of our knowledge, to date, no published studies have examined PTG among frontline healthcare providers during pandemic in limited resource settings such as Kosovo. Gaining a more comprehensive understanding of the potential positive psychological impact of COVID-19 on healthcare providers is essential to developing interventions that would help manage and minimize the consequences of mental health related to pandemic. Therefore, this study sought to provide insight into PTG levels among healthcare providers in Kosovo, following the adversity of the COVID-19 outbreak.

Our study tests the following two hypotheses. First, based on the PTG theoretical model (Tedeschi and Calhoun, 2004; Calhoun and Tedeschi, 2006; Calhoun et al., 2010), coping skills will moderate the relationship between PTG and mental health. And second, the COVID-19 outbreak will have a negative impact on the mental health of healthcare providers.

## Materials and Methods

### Design and Setting

This study employed a cross-sectional and correlational research design to explore the impact of coping skills in developing PTG among healthcare providers.

### Participants

For the sample of the study were recruited physicians, nurses and other healthcare providers working in public health facilities in Kosovo. The present study comprised a sample of 691 healthcare providers (59% female), with an average age of 41.6 years (*SD* = 10.79). Most participants were employed as nurses (*n* = 550), and the rest as physicians (*n* = 138). Mean age of work experience was 16 years (*SD* = 11.31). Over half of the participants had been infected with COVID-19 (*n* = 397) while attending to COVID-19 patients.

### Instruments

#### Socio-Demographic Questionnaire

A self-developed questionnaire was designed by authors to gather sociodemographic data (including age, sex, level of education, if they have been infected with COVID-19, severity of the symptoms they experienced while passing COVID-19 infection, employment status, medical units they work, hours of working and assisting patients with COVID-19 and any participation in psychosocial support programs).

#### General Health Questionnaire-28

GHQ-28 used for general health of participants, requests participants to indicate how their health in general has been over the past few weeks. The questionnaire consists of four sub-scales that measure public health (somatic symptoms, anxiety and insomnia, social dysfunction and depression), and each scale consists of 7 questions. GHQ scoring methods are based on the Likert scale (0 − 1 − 2 − 3), and a low score indicates a better mental state. This questionnaire has shown an excellent value for Cronbach’s Alpha (α = 0.95) (Shayan et al., 2015). According to the Goldberg which say that participants with total scores of 23 or below should be classified with clinical symptoms, while participants with scores > 24 may be classified with clinical symptoms, so Goldberg recommended that each researcher derive a cut-off score based on the mean of their respective sample.

Descriptive data of GHQ28 shows that Somatic Symptoms has *N* = 546 and *M* = 6.77, Anxiety *N* = 674 and *M* = 6.55, Social dysfunction with *N* = 670 and *M* = 8.76, Depression with *N* = 681 and *M* = 4.48 and GHQ in total *N* = 528 with *M* = 26.33.

#### Coping Skills

Coping Skills questionnaire assesses cognitive, emotional, and behavioral methods of dealing with problems. Some items, focusing on cognitive and emotional approaches, were adapted from Holahan and Moos’s (1987) widely used Coping Strategies Scale (items 2, 3, and 4 below), while other cognitive and emotional items were original (1, 5, 6, and 8). The remainder of the items were adapted from Spitzberg and Cupach’s (2008) framework for assessing coping in response to stalking. The total score can be a sum or mean of all the items. Higher scores indicate higher levels of coping. For this study, Cronbach’s alpha for the Coping Skills questionnaire was0.84.

#### Post-traumatic Growth Inventory

The PTGI is an instrument consisting of 21-item, assessing positive outcomes reported by persons who have experienced traumatic events. Its scale includes factors of New Possibilities, Relating to Others, Personal Strength, Spiritual Change, and Appreciation of Life. The PTGI is a 6-point scale self-report measure (0 = I did not experience this change because of my crisis to 5 = I experienced this change to a very great degree because of my crisis). Intermediate scores were given for a very small degree (l), a small degree (2), a moderate degree (3), and a great degree (4). Respondents are asked to indicate the degree to which the statements were true for them, or the change occurred in their life because of their crisis. The summation of all 21 items yielded a total growth score which can range from 0 to 105. Higher scores were indicative of greater growth. Tedeschi and Calhoun (1996) reported the following psychometric data: internal consistency = 0.90, Pearson’s product–moment correlation between the five factors ranged from *r* = 0.27 to 0.52 and test–retest correlation over a 4-week period was 0.79. In the present study, Cronbach’s alpha for the PTGI total score was 0.92 and ranged from 0.88 to 0.93 for subscale scores.

### Procedures

Data collection for the present study was conducted during January 2021. The sample covered all regions in Kosovo and also covers the level of the health system who offered services. First, the study and procedures were approved by the ethics committee of Heimerer University, then permission was obtained from each medical facility. Participants were recruited in medical facilities, and they were contacted face-to-face. Once the objectives and procedures of the study were explained, participants were asked to sign an informed consent form. All participants were assured of their confidentiality, and all of them took part voluntarily. They completed a self-administered questionnaire for study variables including sociodemographic attributes. Since the questionnaires were completed in the context of work during the COVID-19 pandemics, they were filled out in the offices of healthcare providers under safety anti-covid measures. It took up to 20 min to complete the questionnaires. The data collectors were local language speakers.

### Data Analysis

Data analyses were performed using packages stats and dplyr in R Environment, version 4.1.0. We were interested in the presence of PTG and its predictors, to create a basis for further exploration. Missing data was below 5 percent (<5%). The associations of study criterion variables (PTG and the five related subscales) were estimated with several study predictor variables (severity of mental health symptoms, severity of symptoms of those who have survived COVID-19 previously, direct contact with patients, hours spent with patients with COVID-19, and coping). Welch pairwise *t*-test was used to analyze mental health, PTG scales and coping skills differences according to COVID-19 infection, direct contact with patients and gender, with Bonferroni method to account for *p*-value inflation. To compare mental health clinical and nonclinical score distributions according to severity of COVID-19 symptoms, a chi square test was conducted. Due to observed non-normality of the variables, Kruskal Wallis tests were performed to test for differences in PTG scores based on symptom severity. Lastly, to determine the role of coping skills, a mediation model (Baron and Kenny, 1986; Agler and De Boeck, 2017; Bono et al., 2017) was tested, through a two-step bootstrapping method (Preacher and Hayes, 2004) with coping skills as mediator of the relationship between mental health, as measured by GHQ total score, and post-traumatic growth, measured by PTGI total score.

## Results

A considerable proportion of participants reported symptoms above the clinical cut off across all scales. Participants reported fewer clinical symptoms for the four GHQ subscales (Table 1) except somatic symptoms. 44% reported clinical symptoms for the anxiety subscale, 26% for depression, 50% for somatic symptoms, and 39% for social dysfunction.

**TABLE 1:** Self-reported mental health symptoms as measured by GHQ.

	**Anxiety (*n*)**	**Depression (*n*)**	**Somatic symptoms (*n*)**	**Social dysfunction (*n*)**
Clinical symptoms	299	175	274	260
Non-clinical symptoms	375	506	272	410

Table 2 summarized subgroup comparisons across key variables. For GHQ subscales, there was a significant difference for symptom severity on anxiety (χ^2^ = 8.08, *p* < 0.01). Kruskal Wallis analysis showed no statistical differences for all PTG subscales according to symptom severity, and no statistical differences for coping skills (χ^2^ = 59.12, *p* = 0.403).

**TABLE 2:** Mental health, PTG and coping skills group comparisons with reported *t*-test, Kruskal Wallis and chi square tests.

		**Infected with COVID-19**		***t or X***		**Symptom severity**			***t or X***		**Gender**		***t or X***		**Direct Patient Contact**		***t or X***	
		**Yes**	**No**		***p***	**Mild symptoms**	**No symptoms**	**Severe symptoms**		***p***	**Female**	**Male**		***p***	**Yes**	**No**		***p***
Anxiety	Non-clinical	160	198	5.80	*	63	126	28	8.08	* *	212	162	1.42	0.233	233	140	9.41	* *
	Clinical	102	189			39	123	42			184	115			221	48		
Depression	Non-clinical	200	283	0.71	0.398	77	189	44	4.34	0.114	288	217	2.32	0.127	342	163	0.04	0.945
	Clinical	65	109			25	64	26			112	63			118	55		
Somatic symptoms	Non-clinical	117	138	13.62	* * *	48	85	20	5.69	0.057	149	123	16.50	* * *	156	116	9.64	* *
	Clinical	188	80			44	121	40			197	77			184	88		
Social dysfunction	Non-clinical	175	229	2.35	0.124	53	154	35	5.52	0.063	248	162	1.14	0.283	256	152	5.71	*
	Clinical	89	157			50	93	35			145	114			194	66		
PTG *(M* = 47.13)																		
Appreciation of life (Mean)		5.94	6.96	2.87	* *	6.77	7.09	7.11	24.19	0.061	6.58	6.36	0.64	0.520	6.86	5.74	3.26	* * *
Spiritual change (Mean)		5.09	4.45	2.50	*	5.1	5.15	4.91	14.41	0.154	4.88	4.69	0.74	0.453	5.03	4.35	2.65	* * *
Personal strength (Mean)		8.89	9.97	2.30	*	10.02	10	9.64	27.71	0.116	9.65	9.33	−0.61	0.496	10.31	7.94	5.14	* * *
New possibilities (Mean)		10.13	11.44	2.33	*	11.61	11.81	10.82	34.72	0.093	11.11	10.6	0.91	0.359	11.75	9.22	4.68	* * *
Relation to others (Mean)		14.52	16	1.83	0.068	16.32	16.23	15.34	28.3	0.78	15.66	15.03	0.77	0.436	16.81	12.57	5.52	* * *
Coping skills (mean)		36.85	37.11	0.37	0.707	36.9	37.7	35.6	59.12	0.40	37.32	35.97	1.99	*	36.68	36.92	−0.33	0.739

***p < 0.00; **p < 0.01; *p < 0.05.

Self-reported clinical somatic symptoms (χ^2^ = 13.62, *p* < 0.001) and anxiety (χ^2^ = 5.80, *p* < 0.01) differed significantly for participants who experienced COVID-19 infection as opposed to those who didn’t. Results also showed statistical differences across all PTG subscales except relation to others when comparing participants who experienced COVID-19 infection and those who did not. No significant difference was found for coping skills.

While women had more self-reported clinical symptoms than men for four GHQ subscales, only the somatic symptoms differed significantly by gender, χ^2^ = 16.02, *p* < 0.001. Women also had significantly higher coping skills scores than men (*t* = 1.99, *p* < 0.05). No significant difference was found between genders for the post-traumatic growth subscales.

To observe predictor variables for PTG scores, we ran a hierarchical regression analysis with three models, adding variables stepwise to observe variance change. Results in Table 3 show the period of attending to COVID-19 patients significantly predicted PTG in the three models, however the adjusted R2 value did not show changes across each step.

**TABLE 3:** Hierarchical regression results for predictors of experience, direct contact with COVID-19 patients, and period spend attending to COVID-19 patients.

**Predictor variables**	***B***	***Beta***	***t***
**Model 1**			
Period of attending to patients	1.10	0.15	3.28*
**Model 2**			
Period of attending to patients	0.79	0.11	0.04*
Direct contact with patients	–8.95	–0.08	0.12
**Model 3**			
Period of attending to patients	0.89	0.12	0.03*
Direct contact with patients	–9.13	–0.08	0.11
Work experience	–0.08	–0.03	0.54

*Adj. R^2^ = 0.02 for Model 1, p < 0.05; Adj. R^2^ = 0.03 for Model 2, p < 0.05; Adj.*

*R^2^ = 0.02 for Model 3.*

*p < 0.05.

### Mediation Analysis

The mediation analysis showed a significant indirect effect of coping skills on the relationship between mental health and post-traumatic growth (Standardized coefficient = 0.022, 95% CI = [−0.38, −0.11]). Coping skills also significantly predicted post-traumatic growth (Standardized coefficient = −0.10, 95% CI = [0.01, 0.04]). However, there was no significant direct effect between mental health and post-traumatic growth (Standardized coefficient = 0.007, CI = [−0.03, 0.04]).

**FIGURE 1:**
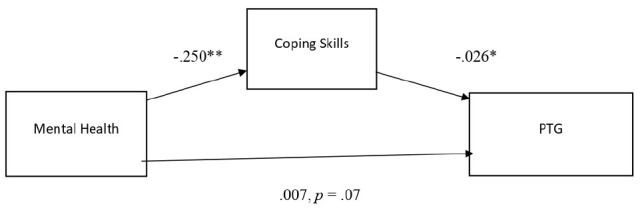
Standardized regression coefficients for the relationship between mental health as measured by GHQ and post-traumatic growth as mediated by coping skills.

After the bootstrapping method was applied, the significant indirect effect of coping skills was confirmed. Figure 1 shows the standardized regression estimates in the model relationship.

Coping skills alone predicted PTG, and mental health alone predicted coping skills. However, mental health did not significantly predict post-traumatic growth, hence no total effect was observed. The results are considered insufficient to determine full mediation in the model.

## Discussion

This study aimed to assess the extent of PTG among a sample of healthcare providers, and to further understand both positive and negative psychological consequences of the current global COVID-19 pandemic, by examining the relationship between PTG, mental health symptomatology and dispositional factors such as coping.

The study revealed alarming data on how COVID-19 outbreak has had an impact on the mental health of healthcare providers. As shown in the Table 1, a considerable proportion of participants reported anxiety symptoms, social dysfunction, depression, with somatic symptoms leading to the escalation. Potential explanations point to the new conditions presented by the pandemic situation, including social withdrawal, excessive hand washing, use of hand sanitizers, protective masks, and medication. Increased focus on protective hygiene behaviors may be linked to the observed somatic symptoms, whereas withdrawal and added work hours may contribute to anxiety. This was particularly true at the time the study was conducted, when also vaccines were not still available as found in Luo et al. (2020) study as well. This may explain why depressive symptoms were less present in the results. Our findings are consistent with recent studies, showing that healthcare providers are at great risk of mental consequences while providing care to patients during COVID-19 pandemic (Shaukat et al., 2020), with high prevalence of somatization associated with medical service-related factors (Song et al., 2021).

The difference on mental health symptoms by gender was found in four subscales and significantly differing particularly in reported somatic symptoms as shown in Table 2. Similar data were found in Gallopeni et al. (2020) study measuring anxiety and depression in healthcare providers in Kosovo, where female healthcare providers have reported almost 20% more anxiety symptoms and 13% more depressive symptoms than their male counterparts’ men. In addition to gender characteristics and etiological research data, this might be influenced by the social norms where women including those employed take more responsibility for childcare, which in turn may have influenced the more pronounced onset of symptoms.

Our findings show low mean scores on PTG. Comparative to previous findings performed in similar settings, the PTG scores of our study lie on mid to high range among samples of healthcare providers similar to Cui et al. (2021) study and is low when compared to firefighters (Yang and Ha, 2019) or to students in the general population (Zeng et al., 2021). A possible explanation could be the time when the study was carried out, as the healthcare providers were still facing tremendous life-threatening situations. Nevertheless, the results showed statistical differences across all PTG subscales except relation to others when comparing participants who experienced COVID-19 infection as opposed to those who did not, as shown in Table 2. In line with other studies (Lau et al., 2006; Bridgland et al., 2021; Huecker et al., 2021; Hyun et al., 2021); COVID-19 as severe acute respiratory syndrome appears to have been experienced as a harmful and traumatic stressful event by healthcare providers, eliciting post-traumatic stress responses. However, overcoming the trauma seems to lead them to constructive outcomes, underlining that PTG can occur after infectious diseases. Upon closer investigation, Relating to Others was the highest scoring PTGI factor, with New Possibilities as second. As a tendency to find meaning in facing this life-threatening disease, healthcare providers seem to change their sense of relationship, resulting in building closer bond with their family members and friends and a willingness to help others, as noted in other studies (Khanjani et al., 2017; Rzeszutek, 2017; Tamiolaki and Kalaitzaki, 2020; Sun et al., 2021) when social support became an important source in coping with challenging events.

Consistent with previous studies (Smith et al., 2008; Lelorain et al., 2010; Rajandram et al., 2011; He et al., 2013; Kunz et al., 2018; Yan et al., 2021), the effect of mental health on PTG was found to be mediated by coping skills in the sample we examined. The link between experiencing COVID-19 infection and the extent of PTG experience indicates that growth may take place not as a direct result of trauma, but a result of the individual’s attempt to adapt to the new post-traumatic reality, as highlighted by Tedeschi et al. (1998). Our findings are congruent with those of other studies (Lelorain et al., 2010; Rajandram et al., 2011; Mesidor and Sly, 2019) suggesting that high scores on adaptive coping strategies is associated with better ability to cope with stressful situations and higher scores on PTG. Coping strategies appear to be influencing factors in well-being (Freire et al., 2016), resilience (Craparo et al., 2018) and post-traumatic growth (as highlighted in this study), which should be further investigated in a tailored study, based on specific events.

Women had significantly higher coping skills scores than men. Similar findings were found in a recent study of Graves et al. (2021) when testing for gender differences in coping and perceived stress among college students. Women are more likely to use emotion-focused strategies such as distraction, and emotional and religious support than men (Park et al., 2020) and more likely to seek instrumental/emotional support, attempt cognitive restructuring, and use spirituality as a “grounding” strategy (Kolakowsky-Hayner et al., 2021). These findings should be further investigated to test whether personality traits have an important role to play in relation to gender difference. While, when it comes to PTG, gender roles did not differentiate for post-traumatic growth. This result supports the finding of Barlow and Hetzel-Riggin (2018) study. Although further investigation is needed to validate our findings and reach a reliable conclusion, the present study results are still meaningful as they show that levels of mental health exacerbation may not playing a conclusive role in determining levels of PTG, if coping mechanism are in place.

## Limitations of the Study

Some limitations must be considered. First, this study was conducted in the early stages of the COVID-19 pandemic when vaccines were not available, and it may reflect on the data regarding the prevalence of mental health symptoms and the extent of post-traumatic growth. Second, self-report measures were used as the primary source of data collection, and are therefore based on perceived capacities, which may lead to the deviation of self-choice. Third, Coping Skills questionnaire due to time constrains, was not standardized; it was only translated and back translated into Albanian. Fourth, the nature of the cross-sectional design was one of the limitations as it limited the course of PTG, it could not reflect the causal-effect relationship. PTG could be related to any pre-trauma events experienced by the participants, and it was not possible to cue measure solely to the experiences of COVID-19. Fifth, this study focused only on quantitative data, lacking triangulation with other methods such as qualitative methods, which should be remedied in future research to give an in-depth insight of PTG experiences of the participants.

## Recommendations

Our findings indicate an urgent need for psychological intervention to recover from mental health struggles and guidelines on how to cope with COVID-19 for this population. The development and implementation of interventions to minimize COVID-19-related mental health consequences and to foster PTG among healthcare providers could be highly beneficial in pandemic response work: providing continues psychological support to healthcare providers, by helping them identify and activate adaptive coping strategies that can lead them to positive life change, regardless the adversity of the COVID-19 outbreak. Mental health care providers should recognize positive changes and growth among their colleagues and use them as a source for improving their skills when facing the negative effects of COVID-19 infection. The growth in relationships exemplified the importance of social support and relationships in the process of facing trauma, which should be considered when developing PTG interventions. The study results fill the gap in the study area of positive changes during the pandemics, by providing baseline information for other researchers to conduct similar studies by considering the limitations of this study.

## Data Availability Statement

The raw data supporting the conclusions of this article will be made available by the authors, without undue reservation.

## Ethics Statement

The studies involving human participants were reviewed and approved by the Heimerer College. The participants provided their written informed consent to participate in this study.

## Author Contributions

All authors listed have made a substantial, direct, and intellectual contribution to the work, and approved it for publication.

## Conflict of Interest

The authors declare that the research was conducted in the absence of any commercial or financial relationships that could be construed as a potential conflict of interest.

## Publisher’s Note

All claims expressed in this article are solely those of the authors and do not necessarily represent those of their affiliated organizations, or those of the publisher, the editors and the reviewers. Any product that may be evaluated in this article, or claim that may be made by its manufacturer, is not guaranteed or endorsed by the publisher.

## References

[B1] Agler R. De Boeck P. (2017). On the interpretation and use of mediation: multiple perspectives on mediation analysis. *Front. Psychol.* 8:1984. 10.3389/fpsyg.2017.01984 29187828 PMC5694788

[B2] Barello S. Palamenghi L. Graffigna G. (2020). Burnout and somatic symptoms among frontline healthcare professionals at the peak of the Italian COVID-19 pandemic. *Psychiatry Res.* 290:113129. 10.1016/j.psychres.2020.113129 PMC7255285 32485487

[B3] Barlow M. R. Hetzel-Riggin M. D. (2018). Predicting posttraumatic growth in survivors of interpersonal trauma: gender role adherence is more important Than gender. *Psychol. Men Masc.* 19 446–456. 10.1037/men0000128

[B4] Baron R. M. Kenny D. A. (1986). The moderator-mediator variable distinction in social psychological research: conceptual, strategic, and statistical considerations. *J. Pers. Soc. Psychol.* 51 1173–1182. 10.1037/0022-3514.51.6.1173 3806354

[B5] Bono R. Blanca M. J. Arnau J. Gómez-Benito J. (2017). Non-normal distributions commonly used in health, education, and social sciences: a systematic review. *Front. Psychol.* 8:1602. 10.3389/fpsyg.2017.01602 28959227 PMC5603665

[B6] Bridgland V. Moeck E. K. Green D. M. Swain T. L. Nayda D. M. Matson L. A. (2021). Why the COVID-19 pandemic is a traumatic stressor. *PLoS One* 16:e0240146. 10.1371/journal.pone.0240146 33428630 PMC7799777

[B7] Calhoun L. G. Cann A. Tedeschi R. G. (2010). “The posttraumatic growth model: sociocultural considerations,” in *Posttraumatic Growth and Culturally Competent Practice: Lessons Learned from Around the Globe*, eds Weiss T. Berger R. (Hoboken, NJ: John Wiley & Sons), 1–14.

[B8] Calhoun L. G. Tedeschi R. G. (2006). “The foundations of posttrau-matic growth: an expanded framework,” in *Handbook of Posttraumatic Growth: Research and Practice*, eds Calhoun L. G. Tedeschi R. G. (Mahwah, NJ: Lawrence Erlbaum), 3–23.

[B9] Chopra V. Toner E. Waldhorn R. Washer L. (2020). How should U.S. hospitals prepare for Coronavirus disease 2019 (COVID-19)? *Ann. Intern. Med.* 172 621–622. 10.7326/M20-0907 32160273 PMC7081177

[B10] Constant E. (2020). Complications neuropsychiatriques de la COVID-19 [Neuropsychiatric complications of COVID-19]. *Rev. Med. Liege* 75 119–122. 33211433

[B11] Craparo G. Magnano P. Zapparrata M. V. Gori A. Costanzo G. Pace U. (2018). Coping, attachment style and resilience: the mediating role of alexithymia. *Mediterr. J. Clin. Psychol.* 6 1–30. 10.6092/2282-1619/2018.6.1773 31160582

[B12] Cui P. P. Wang P. P. Wang K. Ping Z. Wang P. Chen C. (2021). Post-traumatic growth and influencing factors among frontline nurses fighting against COVID-19. *Occup. Environ. Med.* 78 129–135. 10.1136/oemed-2020-106540 33060188

[B13] Freire C. Ferradás M. D. Valle A. Núñez J. C. Vallejo G. (2016). Profiles of psychological well-being and coping strategies among university students. *Front. Psychol.* 7:1554. 10.3389/fpsyg.2016.01554 27790168 PMC5062019

[B14] Gallopeni F. Bajraktari I. Selmani E. Tahirbegolli I. A. Sahiti G. Muastafa A. (2020). Anxiety and depressive symptoms among healthcare professionals during the Covid-19 pandemic in Kosovo: a cross sectional study. *J. Psychosom. Res.* 137:110212. 10.1016/j.jpsychores.2020.110212 32781265 PMC7403847

[B15] Girma A. Ayalew E. Mesafint G. (2021). Covid-19 pandemic-related stress and coping strategies among adults with chronic disease in Southwest Ethiopia. *Neuropsychiatr. Dis. Treat.* 17 1551–1561. 10.2147/NDT.S308394 34045857 PMC8144169

[B16] Graves B. S. Hall M. E. Dias-Karch C. Haischer M. H. Apter C. (2021). Gender differences in perceived stress and coping among college students. *PLoS One* 16:e0255634. 10.1371/journal.pone.0255634 34383790 PMC8360537

[B17] Gupta B. Sharma V. Kumar N. Mahajan A. (2020). Anxiety and sleep disturbances among health care workers during the COVID-19 pandemic in India: cross-sectional online survey. *JMIR Public Health and Surveill.* 6:e24206. 10.2196/24206 33284784 PMC7758087

[B18] He L. Xu J. Wu Z. (2013). Coping strategies as a mediator of posttraumatic growth among adult survivors of the Wenchuan earthquake. *PLoS One* 8:e84164. 10.1371/journal.pone.0084164 24386345 PMC3873973

[B19] Helgeson V. S. Reynolds K. A. Tomich P. L. (2006). A meta-analytic review of benefit finding and growth. *J. Consult. Clin. Psychol.* 74, 797–816. 10.1037/0022-006X.74.5.797 17032085

[B20] Holahan C. J. Moos R. H. (1987). Personal and contextual determinants of coping strategies. *J. Pers. Soc. Psychol.* 52 946–955. 10.1037/0022-3514.52.5.946 3585703

[B21] Huecker M. Shreffler J. Danzl D. (2021). COVID-19: optimizing healthcare provider wellness and posttraumatic growth. *Am. J. Emerg. Med.* 46 693–694. 10.1016/j.ajem.2020.08.066 32917427 PMC7449892

[B22] Hyun H. S. Kim M. J. Lee J. H. (2021). Factors associated with post-traumatic growth among healthcare workers who experienced the outbreak of MERS virus in South Korea: a mixed-method study. *Front. Psychol.* 12:541510. 10.3389/fpsyg.2021.541510 33967871 PMC8100316

[B23] Kalaitzaki A. E. Tamiolaki A. Rovithis M. (2020). The healthcare professionals amidst COVID-19 pandemic: a perspective of resilience and posttraumatic growth. *Asian J. Psychiatry* 52:102172. 10.1016/j.ajp.2020.102172 32426063 PMC7227560

[B24] Khanjani M. S. Younesi S. J. Khankeh H. R. Azkhosh M. (2017). Exploring facilitators of post-traumatic growth in patients with spinal cord injury: a qualitative study. *Electron. Phys.* 9 3544–3553. 10.19082/3544 PMC5308493 28243405

[B25] Kolakowsky-Hayner S. A. Goldin Y. Kingsley K. Alzueta E. Arango-Lasprilla J. C. Perrin P. B. (2021). Psychosocial impacts of the COVID-19 quarantine: a study of gender differences in 59 countries. *Medicina* 57:789. 10.3390/medicina57080789 PMC8400641 34440995

[B26] Kunz S. Joseph S. Geyh S. Peter C. (2018). Coping and posttraumatic growth: a longitudinal comparison of two alternative views. *Rehabil. Psychol.* 63 240–249. 10.1037/rep0000205 29878829

[B27] Lau J. T. Yang X. Tsui H. Y. Pang E. Wing Y. K. (2006). Positive mental health-related impacts of the SARS epidemic on the general public in Hong Kong and their associations with other negative impacts. *J. Infect.* 53, 114–124. 10.1016/j.jinf.2005.10.019 16343636 PMC7132442

[B28] Lai J. Ma S. Wang Y. Cai Z. Hu J. Wei N. (2020). Factors associated with mental health outcomes among health care workers exposed to Coronavirus disease 2019. *JAMA Netw. Open* 3:e203976. 10.1001/jamanetworkopen.2020.3976 32202646 PMC7090843

[B29] Lee S. A. (2020). Coronavirus anxiety scale: a brief mental health screener for COVID-19 related anxiety. *Death Stud.* 44 393–401. 10.1080/07481187.2020.1748481 32299304

[B30] Lelorain S. Bonnaud-Antignac A. Florin A. (2010). Long term posttraumatic growth after breast cancer: prevalence, predictors and relationships with psychological health. *J. Clin. Psychol. Med. Settings* 17 14–22. 10.1007/s10880-009-9183-6 20082122

[B31] Luo M. Guo L. Yu M. Jiang W. Wang H. (2020). The psychological and mental impact of coronavirus disease 2019 (COVID-19) on medical staff and general public–a systematic review and meta-analysis. *Psychiatry Res.* 291:113190. 10.1016/j.psychres.2020.113190 32563745 PMC7276119

[B32] Manning-Jones S. de Terte I. Stephens C. (2015). Vicarious posttraumatic growth: a systematic literature review. *Int. J. Wellbeing* 5 125–139. 10.5502/ijw.v5i2.8 32285354

[B33] Mesidor J. K. Sly F. K. (2019). Religious coping, general coping strategies, perceived social support, PTSD symptoms, resilience, and posttraumatic growth among survivors of the 2010 earthquake in Haiti. *Mental Health Relig. Cult.* 22 130–143. 10.1080/13674676.2019.1580254

[B34] Neto M. L. R. Almeida H. G. Esmeraldo J. D. a. Nobre C. B. Pinheiro W. R. de Oliveira C. R. T. (2020). When health professionals look death in the eye: the mental health of professionals who deal daily with the 2019 coronavirus outbreak. *Psychiatry Res.* 288:112972. 10.1016/j.psychres.2020.112972 PMC7152886 32302817

[B35] National Institute of Public Health [NIPH] (2020). *National Institute of Public Health of Kosova. (Press release).* Prishtina: NIPH.

[B36] Park C. L. Russell B. S. Fendrich M. Finkelstein-Fox L. Hutchison M. Becker J. (2020). Americans’ COVID-19 stress, coping, and adherence to CDC guidelines. *J. Gen. Intern. Med.* 35 2296–2303. 10.1007/s11606-020-05898-9 32472486 PMC7259430

[B37] Prati G. Pietrantoni L. (2009). Optimism, social support, and coping strategies as factors contributing to posttraumatic growth: A meta-analysis. *J. Loss Trauma* 14, 364–388. 10.1080/15325020902724271

[B38] Preacher K. Hayes A. (2004). SPSS and SAS procedures for estimating indirect effects in simple mediation models. *Behav. Res. Methods Instrum. Comput.* 36 717–731. 10.3758/BF03206553 15641418

[B39] Qiu D. Yu Y. Li R. Q. Li Y. L. Xiao S. Y. (2020). Prevalence of sleep disturbances in Chinese healthcare professionals: a systematic review and meta-analysis. *Sleep Med.* 67 258–266. 10.1016/j.sleep.2019.01.047 31040078

[B40] Rajandram R. K. Jenewein J. McGrath C. Zwahlen R. A. (2011). Coping processes relevant to posttraumatic growth: an evidence-based review. *Support. Care Cancer* 19 583–589. 10.1007/s00520-011-1105-0 21298449 PMC3069319

[B41] Rodríguez-Rey R. Palacios A. Alonso-Tapia J. Pérez E. Álvarez E. Coca A. (2017). Posttraumatic growth in pediatric intensive care personnel: dependence on resilience and coping strategies. *Psychol. Trauma* 9 407–415. 10.1037/tra0000211 27929306

[B42] Rogan C. Fortune D. G. Prentice G. (2013). Post-traumatic growth, illness perceptions and coping in people with acquired brain injury. *Neuropsychol. Rehabil.* 23 639–657. 10.1080/09602011.2013.799076 23701407

[B43] Rzeszutek M. (2017). Social support and posttraumatic growth in a longitudinal study of people living with HIV: the mediating role of positive affect. *Eur. J. Psychotraumatol.* 8:1412225. 10.1080/20008198.2017.1412225 PMC5738637 29296241

[B44] Sears S. R. Stanton A. L. Danoff-Burg S. (2003). The yellow brick road and the emerald city: benefit finding, positive reappraisal coping and posttraumatic growth in women with early-stage breast cancer. Health psychology: official *J. Div. Health Psychol. Am Psychol.* 22, 487–497. 10.1037/0278-6133.22.5.487 14570532

[B45] Shaukat N. Ali D. M. Razzak J. (2020). Physical and mental health impacts of COVID-19 on healthcare workers: a scoping review. *Int. J. Emerg. Med.* 13:40. 10.1186/s12245-020-00299-5 32689925 PMC7370263

[B46] Shayan Z. Pourmovahed Z. Najafipour F. Abdoli A. M. Mohebpour F. Najafipour S. (2015). Factor structure of the general health questionnaire-28 (GHQ-28) from infertile women attending the Yazd Research and Clinical Center for Infertility. *Int. J. Reprod. Biomed.* 13 801–808. 27141541 PMC4827513

[B47] Siegel K. Schrimshaw E. W. Pretter S. (2005). Stress-related growth among women living with HIV/AIDS: examination of an explanatory model. *J. Behav. Med.* 28, 403–414. 10.1007/s10865-005-9015-6 16179979

[B48] Smith B. W. Dalen J. Bernard J. F. Baumgartner K. B. (2008). Posttraumatic growth in non-Hispanic White and Hispanic women with cervical cancer. *J. Psychosoc. Oncol.* 26 91–109. 10.1080/07347330802359768 19042274

[B49] Song X. Zhou Y. Rao W. Zhang X. (2021). Comparison of prevalence and risk factors of somatization between Chinese health care workers and non-health care workers during COVID-19 outbreak. *BMC Psychiatry* 21:276. 10.1186/s12888-021-03294-z 34059033 PMC8165950

[B50] Spitzberg B. Cupach W. (2008). “Managing unwanted pursuit,” in *Studies in Applied Interpersonal Communication*, ed. Motley M. (Thousand Oaks, CA: Sage), 3–25. 10.4135/9781412990301.d3

[B51] Sun W. Chen W.-T. Zhang Q. Ma S. Huang F. Zhang L. (2021). Post-traumatic growth experiences among COVID-19 confirmed cases in China: a qualitative study. *Clin. Nurs. Res.* 30 1079–1087. 10.1177/10547738211016951 34018405 PMC9167671

[B52] Tamiolaki A. Kalaitzaki A. E. (2020). “That which does not kill us, makes us stronger”: COVID-19 and posttraumatic growth. *Psychiatry Res.* 289:113044. 10.1016/j.psychres.2020.113044 PMC7191294 32388177

[B53] Tedeschi R. G. Calhoun L. G. (1995). *Trauma* & *Transformation: Growing in the Aftermath of Suffering.* Thousand Oaks, CA: Sage Publications, Inc., 10.4135/9781483326931

[B54] Tedeschi R. G. Calhoun L. G. (1996). The posttraumatic growth inventory: measuring the positive legacy of trauma. *J. Traum. Stress* 9 455–471. 10.1007/BF02103658 8827649

[B55] Tedeschi R. G. Calhoun L. G. (2004). Posttraumatic growth: conceptual foundations and empirical evidence. *Psychol. Inq.* 15 1–18.

[B56] Tedeschi R. G. Moore B. A. (2021). Posttraumatic growth as an integrative therapeutic philosophy. *J. Psychother. Integr.* 31 180–194. 10.1037/int0000250

[B57] Tedeschi R. G. Park C. L. Calhoun L. G. (1998). “Posttraumatic growth: conceptual issues,” *Posttraumatic Growth: Positive Changes in the Aftermath of Crisis*, eds Tedeschi R. G. Park C. L. Calhoun L. G. (Lawrence Erlbaum Associates Publishers), 1–22.

[B58] Wang C. Horby P. W. Hayden F. G. Gao G. F. (2020). A novel coronavirus outbreak of global health concern. *Lancet* 395 470–473. 10.1016/S0140-6736(20)30185-9 31986257 PMC7135038

[B59] World Health Organization [WHO] (2020). *Coronavirus Disease 2019 (COVID-19): Situation Report.* Geneva: World Health Organization, 67.

[B60] Wu X. Kaminga A. C. Dai W. Deng J. Wang Z. Pan X. (2019). The prevalence of moderate-to-high posttraumatic growth: a systematic review and meta-analysis. *J. Affect. Disord.* 243 408–415. 10.1016/j.jad.2018.09.023 30268956

[B61] Yan S. Yang J. Ye M. Chen S. Xie C. Huang J. (2021). Post-traumatic growth and related influencing factors in discharged COVID-19 patients: a cross-sectional study. *Front. Psychol.* 12:658307. 10.3389/fpsyg.2021.658307 34122242 PMC8189317

[B62] Yang S. K. Ha Y. (2019). Predicting posttraumatic growth among firefighters: the role of deliberate rumination and problem-focused coping. *Int. J. Environ. Res. Public Health* 16:3879. 10.3390/ijerph16203879 31614945 PMC6843524

[B63] Zeng W. Zeng Y. Xu Y. Huang D. Shao J. Wu J. (2021). The influence of post-traumatic growth on college students’ creativity during the COVID-19 pandemic: the mediating role of general self-efficacy and the moderating role of deliberate rumination. *Front. Psychol.* 12:1264. 10.3389/fpsyg.2021.665973 33935927 PMC8079774

[B64] Zhang C. Yang L. Liu S. Ma S. Wang Y. Cai Z. (2020). Survey of insomnia and related social psychological factors among medical staff involved in the 2019 novel Coronavirus disease outbreak. *Front. Psychiatry* 11:306. 10.3389/fpsyt.2020.00306 32346373 PMC7171048

